# Biosynthesis and Properties of Sulfur-Containing Polyhydroxyalkanoates (PHAs) Produced by Wild-Type Strain *Cupriavidus necator* B-10646

**DOI:** 10.3390/polym15041005

**Published:** 2023-02-17

**Authors:** Natalia O. Zhila, Kristina Yu. Sapozhnikova, Arina V. Berezovskaya, Evgeniy G. Kiselev, Ekaterina I. Shishatskaya, Aleksander D. Vasiliev, Sabu Thomas, Tatiana G. Volova

**Affiliations:** 1 Institute of Biophysics SB RAS, Federal Research Center “Krasnoyarsk Science Center SB RAS”, 50/50 Akademgorodok, 660036 Krasnoyarsk, Russia; 2 Basic Department of Biotechnology, School of Fundamental Biology and Biotechnology, Siberian Federal University, 79 Svobodnyi Av., 660041 Krasnoyarsk, Russia; 3 Department of Medical Biology, School of Fundamental Biology and Biotechnology, Siberian Federal University, 79 Svobodnyi Av., 660041 Krasnoyarsk, Russia; 4 L.V. Kirensky Institute of Physics SB RAS, Federal Research Center “Krasnoyarsk Science Center SB RAS”, 50/38 Akademgorodok, 660036 Krasnoyarsk, Russia; 5 Basic Department of Solid State Physics and Nanotechnology, School of Engineering Physics and Radio Electronics, Siberian Federal University, 26 Kirensky St., 660074 Krasnoyarsk, Russia; 6 International and Interuniversity Centre for Nano Science and Nano Technology, Mahatma Gandhi University, Kottayam 686560, India

**Keywords:** biosynthesis, different precursors, sulfur-containing PHAs, chemical composition, 3MP monomers contents, properties

## Abstract

The study addresses the growth of the wild-type strain *Cupriavidus necator* B-10646 and the synthesis of sulfur-containing polyhydroxyalkanoates (PHA) by this strain on media containing fructose and three different precursors (3-mercaptopropionic acid, 3′,3′-dithiodipropionic acid and 3′,3′-thiodipropionic acid). By varying the concentration and number of doses of the precursors added into the bacterial culture, it was possible to find conditions that ensure the formation of 3-mercaptopropionate (3MP) monomers from the precursors and their incorporation into the C-chain of poly(3-hydroxybutyrate). A series of P(3HB-*co*-3MP) copolymer samples with different content of 3MP monomers (from 2.04 to 39.0 mol.%) were synthesized and the physicochemical properties were studied. The effect of 3MP monomers is manifested in a certain decrease in the molecular weight of the samples and an increase in polydispersity. Temperature changes are manifested in the appearance of two peaks in the melting region with different intervals regardless of the 3MP content. The studied P(3HB-*co*-3MP) samples, regardless of the content of 3MP monomers, are characterized by equalization of the ratio of the amorphous and crystalline phases and have a close degree of crystallinity with a minimum of 42%, = and a maximum of 54%.

## 1. Introduction

Microbial degradable polyhydroxyalkanoates (PHA) are a promising candidate for replacing synthetic non-degradable plastics derived from oil, the accumulation of which in the biosphere is a global environmental problem [1,2,3,4,5,6]. PHA is a family of polymers of various chemical composition synthesized by prokaryotes on various carbon substrates. The physicochemical properties of these polymers (molecular weight, temperature characteristics, degree of crystallinity, degradation rates in biological media, etc.) and the possibility of processing into specialized products, vary significantly depending on their chemical structure. First, they depend on the structure of the side groups in the polymeric C-chain and on the distance between the ester groups in the molecule [7,8,9,10,11,12,13,14].

The first identified polymer from the PHA class and the most fully characterized and commercially produced is poly(3-hydroxybutyrate) [P(3HB)], which is a highly crystalline isotactic polyester with regular, uniformly oriented (“head-to-tail”) successive units of D-(-)-3-*β*-hydroxybutyric acid [15]. The copolymer of 3-hydroxybutyrate with 3-hydroxyvalerate [P(3HB-*co*-3HV)] became the second most actively studied representative of the PHA family after P(3HB). These copolymers are isodimorphic due to the co-crystallization of the monomers; when the ratio of monomers changes in them, changes in the crystal lattice take place. The inclusion of 3-hydroxyvalerate in the C-chain of 3-hydroxybutyrate significantly affects the crystallization kinetics of the material, and with an increase in its content, the material becomes less crystalline and more elastic [16]. Copolymers of 3-hydroxybutyrate and 4-hydroxybutyrate [P(3HB-*co*-4HB)] are an elastic polymeric material whose elongation at break can be up to 1000%. This is two orders of magnitude higher than that of P(3HB). At a high (over 50 mol.%) content of 4HB monomers in the copolymer, this type of PHA has only one type of crystal lattice and 4HB monomers are not included in the 3HB lattice [17].

Based on the length of the carbon chain, the monomers in PHA have been subdivided into short chain length (SCL) polymers consisting of acids with a C-chain length of three to five carbon atoms; medium chain length (MCL) polymers containing from 6 to 14 carbon atoms; and long chain length (LCL) polymers contain monomers with a C-chain length of C17 and above [18]. The range of known PHAs is expanding over time. Olivera et al., in 2010, proposed to divide PHA into two categories depending on the frequency of occurrence: “usual” and “unusual” [19]. It is proposed to refer to “unusual” PHA and, as a rule, poorly studied various homopolymer and copolymer PHA, including semi-synthetic and synthetic polymers that contain various functional groups and are synthesized from natural monomers or from their chemical derivatives and polymers obtained by modifying polymers, synthesized by microorganisms [19].

The potential possibility of synthesized polymers of various compositions formed by monomers with different C-chain lengths is a particularly valuable property of PHA since depending on the ratio of monomers their basic properties primarily the degree of crystallinity can vary within fairly wide limits [7,8,12,13,20,21]. The variety of possible pathways for the synthesis of PHA [1] in various microorganisms using a wide range of carbon substrates is a real basis for the synthesis of a wide range of polymers of various chemical compositions.

A new class of biopolymers, which are also synthesized by the PHA synthase, were detected relatively recently. When the Gram-negative bacterium *Ralstonia eutropha* was cultivated in the presence of 3-mercaptoalkanoic acids or 3,3′-thiodipropionic acid, copolymers consisting of 3-mercaptoalkanoates and 3HB were synthesized. The constituents were linked by thioester and by oxoester bonds. So far, 3-mercaptopropionic acid (3MP) [22] and 3-mercaptobutyric acid (3MB) [23] have been described as constituents of these novel PTEs.

Polythioesters, which are a class of PHA containing sulfur atoms in the carbon chain, belong to “unusual” and little-studied types of PHAs. Bacterial PTEs are materials with unusual properties, one of which is difficult crystallization processes compared to most known types of PHAs. The melting point of poly(3-mercaptopropionate) [P(3MP)] is close to that of P(3HB), but P(3MP) exhibits higher thermal stability. It has also been shown that copolymers of this type have antibacterial properties [24,25]. The higher thermal stability of P(3MP) is combined with a property that is unusual for the PHA family—the lack of biodegradability. On the other hand, this feature of individual PTEs limits their scope as promising biodegradable polymeric materials in comparison with most known PHAs, but, on the other hand, it opens up new prospects. The bioresistance of PTEs to microbial or enzymatic biodegradation makes them promising thermoplastics to replace petroleum-derived polyolefins in different applications that require material durability, such as construction and the automotive industry, as a component in home building, automotive assembly and photovoltaic elements applications. In addition, the potential use of these types of PHA as a biocompatible antibacterial material in regenerative medicine when biocompatible endoprostheses must function in the body for a long time is discussed [26,27]. It was shown that the P(3MP) homopolymer synthesized by the recombinant *E. coli* strain JM109 is resistant to microbiological degradation, although it was synthesized bacterially [28]. In contrast to the P(3MP) homopolymer, the poly(3-hydroxybutyrate-*co*-3-mercaptopropionate) copolymer [P(3HB-*co*-3MP)] is capable of microbiological biodegradation [29,30].

The analysis of publications showed that a very limited number of scientific teams are engaged in the study of these unusual sulfur-containing PHAs. The leader of the publications and the first to discover the possibility of their synthesis was the team headed by Professor A. Steinbüchel (Germany). There are quite a few works published to date [22,23,25,31,32,33,34,35,36] (Table 1). At the same time, they contain little information about the production culture parameters and, as a rule, low total PHA yields are reported. Various compounds are used as precursors necessary for the synthesis of sulfur-containing monomers: 3-mercaptopropionic acid, 3,3′-dithiodipropionic acid or 3,3′-thiodipropionic acid.

Data on the physicochemical properties of sulfur-containing copolymers are presented fragmentarily in a few publications. The summarized literature data presented in Table 1 show that there is mainly fragmentary information on the molecular weight of individual types of PTE with different content of 3MP monomers; from the temperature characteristics, mainly the melting point is shown and data on the degree of crystallinity are not given in most papers.

The aim of this work is to study the conditions for the synthesis of sulfur-containing copolymers in the culture of the wild-type strain *Cupriavidus necator* B-10646 using various precursors and to reveal the relationship between the composition of monomers and physicochemical properties.

## 2. Materials and Methods

### 2.1. Microorganisms

All experiments were performed with a wild-type strain *Cupriavidus necator* B-10646 registered in the Russian National Collection of Industrial Microorganisms [40].

### 2.2. Culture Medium and Cultivation Conditions

The strain was cultivated in a strong phosphate-buffered solution—the mineral Schlegel medium [41]. The main carbon source was fructose (Panreac, Barcelona, Spain), which was sterilized by membrane filtration (Opticap XL300 Millipore Express SHC filters, Merck, Darmstadt, Germany). To synthesize PHA copolymers, the cell culture was supplemented with precursors of 3-mercaptopropionic, 3,3′-thiodipropionic and 3,3′-dithiodipropionic acids (Sigma-Aldrich, Saint Louis, MO, USA). Cells were grown in the batch culture in the mode previously developed for PHA synthesis [42]. An inoculum was obtained using an Innova^®^ 44 constant temperature incubator shaker (New Brunswick Scientific, Edison, NJ, USA). To prepare the inoculum, the stock culture maintained on agar medium was resuspended culture and grown in 0.5-L glass flasks half-filled with mineral solution with the initial concentration of fructose (10.0–15.0 g/L). The concentration of fructose was determined using the resorcinol method [43]. The indicators of the process of PHA synthesis by bacteria using various precursor substrates necessary for the formation of 3-mercaptopropionate (3MP) monomers were the intracellular content of the polymer (% of CDW) and the concentration of bacterial biomass (X, g/L), which are registered according to the results of the analysis of periodically taken samples.

### 2.3. PHA Recovery from Cell Biomass

To extract PHAs cell biomass was separated from the culture fluid by centrifugation using an AvantyJ-HC centrifuge (BeckmanCoulter, Indianapolis, IN, USA) and dried in LP10R freeze dryer (ilShinBioBase, Dongducheon-si, Korea) to a residual moisture content of 5%. The polymer extraction was performed in two stages. First, the biomass was processed with ethanol to remove lipids and fatty acids. The second stage had the polymer extracted with dichloromethane. The dichloromethane extracts were pooled and evaporated twice using an R/210 V rotary evaporator (Büchi, Flawil, Switzerland). Then, polymer was precipitated with hexane, 1:2. To purified polymer it was redissolved in chloroform several times and precipitated using hexane. The resulting polymer was dried at 40 °C [44].

### 2.4. PHA Chemical Composition

To determine the intracellular content of the copolymer and their composition dry cell biomass, specimens were isolated and purified samples of the polymers were analyzed. The PHA intracellular content and composition were determined by 7890A chromatograph-mass spectrometer (Agilent Technologies, Santa Clara, CA, USA) equipped with a 5975C mass detector (Agilent Technologies, Santa Clara, CA, USA). Purity of the polymer and its composition were determined by chromatography of methyl esters of fatty acids after methanolysis of polymer samples using a chromatograph-mass spectrometer. To carry out the methanolysis procedure, a test tube with a solution containing 1.00 mL of chloroform, 0.85 mL of methanol and 0.15 mL of sulfuric acid and 3.9–4.5 mg of dry cells was heated under a reverse refrigerator for 2 h 40 min at 80 °C [45]. Then, 1.0 mL of distilled water was added. After the phase separation, the lower organic phase containing fatty acid methyl esters was analyzed. Benzoic acid was used as an internal standard for determining total intracellular PHA.

### 2.5. Physicochemical Properties of PHAs

Physicochemical properties of PHAs were examined using size exclusion chromatography, differential scanning calorimetry and X-ray structure analysis; methods and instruments have been described in detail elsewhere [46].

Molecular weight and molecular weight distribution of PHA specimens were examined using a size exclusion chromatography (Agilent Technologies 1260 Infinity, Waldbronn, Germany) equipped with a DB-35MS column. Chloroform was the eluent at a flow rate of 1.0 mL/min with sample concentrations and injection volumes of 5.0 mg/mL and 50.0 μL, respectively, at 40 °C. Calibration was done using polystyrene standards (Agilent Technologies, Santa Clara, CA, USA). Molecular weights (weight average, M_w_, and number average, M_n_) and polydispersity (Ð = M_w_/M_n_) were determined.

To determine thermal properties of PHA specimens thermal analysis was carried out employing a DSC-1 differential scanning calorimeter (Mettler Toledo, Schwerzenbac, Switzerland) and TGA (Mettler Toledo, Schwerzenbac, Switzerland). The crystallization temperature (T_cryst_) was detected by exothermic peaks and the glass transition temperature (T_g_), melting point (T_melt_) and thermal degradation temperature (T_degr_) were determined by endothermic peaks on the thermograms. The thermograms were analyzed using the “STARe v11.0” software (Mettler Toledo, Schwerzenbac, Switzerland).

To determine crystallinity of the synthesized copolymers in the form of films, X-ray structure analysis was carried out using a D8 ADVANCE X-ray powder diffractometer and a VANTEC fast linear detector (Bruker AXS, Karlsruhe, Germany). Crystallinity (C_x_) was detected as the ratio of the area under the radiograph with the subtracted amorphous background to the area without subtracting the background. The Eva program from the diffractometer software application was applied for calculations.

### 2.6. Statistics

Statistical processing of experimental data was carried out by conventional methods using a standard Microsoft Excel software package. Arithmetic means and standard deviations were found. The statistical significance of results was determined using Student’s *t*-test (significance level: *p* ≤ 0.05). Each experiment was performed in triplicate.

## 3. Results and Discussion

The biotechnological synthesis of PHA copolymers is a very complex technological problem. This is due to the fact that it requires the use of additional carbon substrates, the so-called precursor substrates of target monomers, which, as a rule, are toxic for producer strains and reduce their production characteristics. Therefore, it is very difficult to simultaneously obtain a high yield of cell biomass with a high intracellular content of the polymer and the inclusion of monomers with different C-chain lengths. Thus, it is important to select precursors, determine their concentrations that do not inhibit the growth of microorganisms and find the conditions and modes of biosynthesis under which it is possible to PHA with different content of monomers, which can affect the basic properties of polymers.

It is known that 3MP monomer precursors cannot be used by wild-type strains of *C. necator* as the sole carbon source [33]. Therefore, in all experiments, fructose was used as the main source of carbon and different precursors of sulfur-containing monomers.

### 3.1. Study of 3-Mercaptopropionic Acid as a Precursor for the Synthesis of Sulfur-Containing Copolymers in the Culture of Cupriavidus necator B-10646

**Figure 1:**
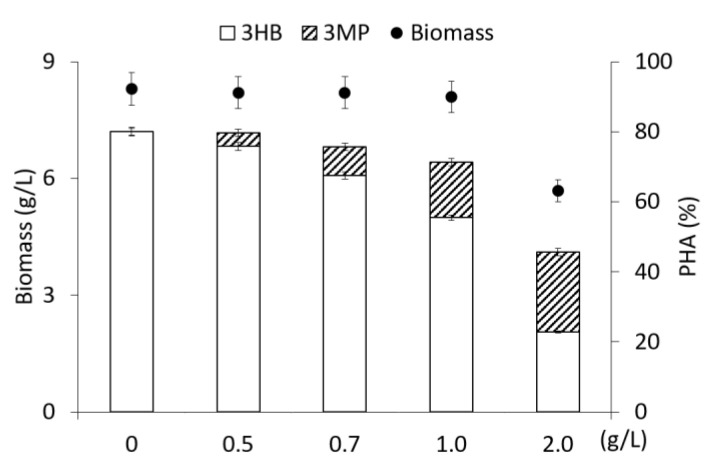
Biomass concentration of *Cupriavidus necator* B-10646, content and composition of PHA in experiments with fructose as the main C-substrate and addition of 3-meraptopropionic acid at various concentrations supplemented into medium on 24 h of cultivation.

The results of the study of the synthesis of sulfur-containing copolymers using 3-mercaptopropionic acid as a precursor at a concentration from 0.5 to 2.0 g/L are shown in Figure 1.

The precursor substrate was supplemented once into the medium at various concentrations during the period of active polymer synthesis (for 24 h of cultivation). The addition of 3-mercaptopropionic acid to the culture at a concentration of up to 1.0 g/L had no inhibitory effect on bacterial growth and polymer synthesis. The bacterial biomass concentration and the intracellular PHA content at the end of the cultivation (72 h) were 8.2 ± 0.1 g/L and 75.0 ± 4.0%, respectively. This is comparable to the performance of the control culture grown only on fructose without the addition of 3-mercaptopropionic acid.

Subsequent increase in the concentration of the precursor substrate to 2.0 g/L inhibited bacterial growth and polymer synthesis. Additionally, biomass concentration and polymer content decreased to 5.7 g/L and 45.6%, respectively. It is known that thiols, which include 3-mercaptopropionic acid, are the cause of oxidative stress in bacterial cells as a result of the induction of reactive oxygen species [47] and 3MP, especially when converted to 3MP-CoA, has an inhibitory effect on the enzymes of reactions *β*-oxidation [48].

Data on the production parameters of the synthesis of such polymers, available in the literature, are very limited. It was shown that the content of the P(3HB-*co*-3MP) copolymer synthesized by *Ralstonia eutropha* H16 (DSM 428) was not high (from 9.1 to 31.3%). At the same time, the parameters of the concentration of bacterial biomass are not reported [22,23]. Regarding the inhibition of this strain growth by 3-mercaptopropionic acid, the authors noted the absence of an inhibitory effect from this precursor at concentrations up to 1.0 g/L. That is consistent with our data for the *C. necator* B-10646 strain.

**Figure 2:**
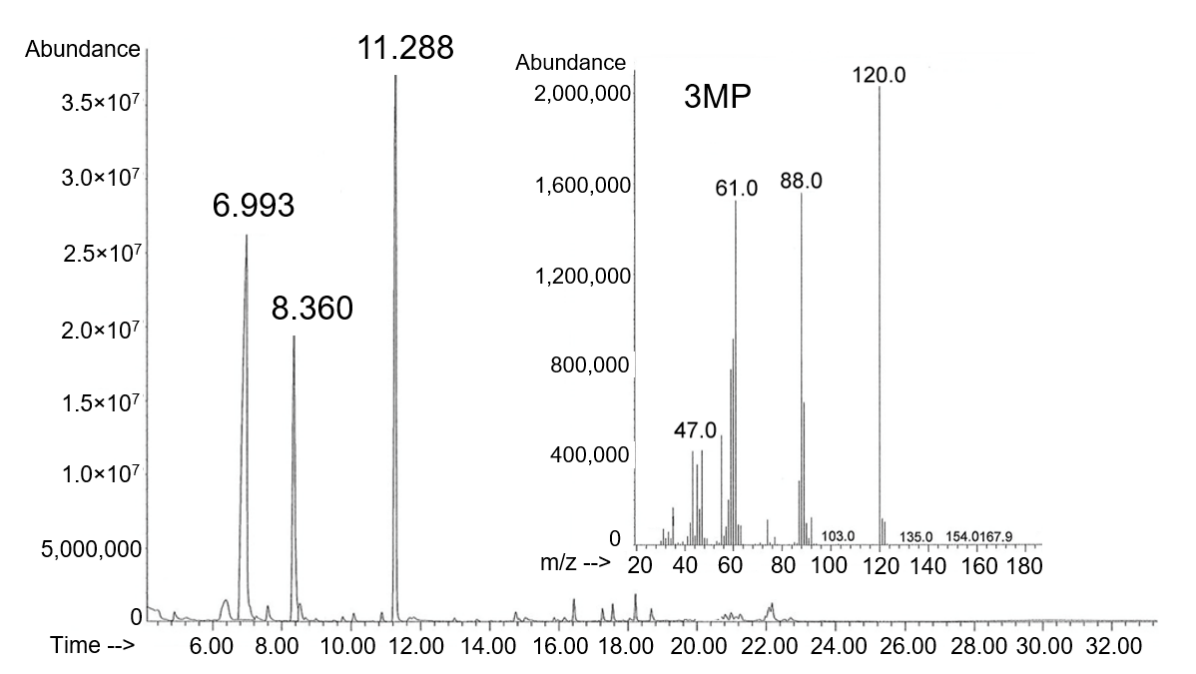
Chromatogram and mass spectra of the P(3HB-*co*-3MP) sample with the content of 3HB and 3MP monomers are, respectively, 84.0 and 16.0 mol.%. The retention times of the monomers are, respectively, 3HB 6.993; 3MP 8.360.

The presence of 3MP monomers in PHA samples synthesized on a medium with the addition of 3-mercaptopropionic acid was confirmed by chromatographic analysis and mass spectra. To determine the composition of PHA samples synthesized by *C. necator* B-10646 on fructose supplemented with 3-mercaptopropionic acid at various concentrations, we analyzed not only the biomass of bacteria, but also polymer samples isolated from cells and purified (Figure 2).

The synthesized samples were two-component P(3HB-*co*-3MP) copolymers with different content of 3-hydroxybutyrate (3HB) monomers (from 50.1 to 95.2 mol.%) and 3-mercaptopropionate (3MP) monomers (from 4.8 mol.% to 49.9 mol.%) (Figure 1). When 3-mercaptopropionic acid was supplemented at concentrations from 0.5 g/L to 1.0 g/L, the content of 3MP monomers in the copolymer increased from 4.9 mol.% to 22.2 mol.%. The maximum content of 3MP monomers (49.9 mol.%) was found after a single addition of the precursor into the medium at a concentration of 2.0 g/L; however, the total polymer content sharply decreased significantly to 45.6%.

The analysis of polymer samples isolated from the cell biomass showed a lower content of 3MP monomers in the copolymer compared to the data obtained from the analysis of the total cell biomass. Thus, when 3-mercaptopropionic acid was added to the culture at a concentration of 0.5 g/L to 1.0 g/L, 3MP inclusions did not exceed 1.96–2.04 mol.%. The maximum content of 3MP monomers (4.5 mol.%), which was not high, was obtained when 2.0 g/L of the precursor was added to the culture. Additionally, high values of the content of 3MP monomers obtained from the analysis of cell biomass samples (in contrast to the analysis of isolated polymer samples) are associated with the detection of 3MP monomers included in the polymer chain and in the intracellular pool of 3MP. It is formed as a result of passive intake of 3-mercaptopropionic acid into cells and not included in the synthesis of 3MP monomers and their incorporation into the polymer chain.

Comparison of these results with published results showed the following: describe similar P(3HB-*co*-3MP) copolymers, in which the content of 3MP monomers was determined not by the direct chromatographic analysis of polymer samples, but by recalculation for the elemental sulfur content in bacterial cells, which was higher (from 26.9 mol.% to 42.5 mol.%) [22,31]. In our opinion, this indirect method for characterizing the inclusion of 3MP monomers in sulfur-containing copolymers, without taking into account the proportion of sulfur bound in sulfur-containing amino acids, is not correct. The authors of [22,31,33,36] noted that, in all cases, with an increase in the proportion of 3MP in the copolymer, a decrease in the total content of the polymer in the cells occurred. A decrease in the total content of the copolymer with an increase in the 3MP content was also shown in the present study (Figure 1). However, the overall yields of the copolymer remained at a high level. Lütke-Eversloh et al., 2002 [31] showed that, when scaling up the cultivation of *R. eutropha* H16 in fed-batch culture in a 28 L agitated fermenter (at 200–400 rpm) and a 30 L stainless steel fermenter (Biostat UD30, B Braun, Biotech International, Melsungen, Germany), bacterial biomass was up to 300 g and isolated polymer was about 70 g. The content of 3MP monomers in the polymer varied over a wide range (from 4.6 mol.% to 42.5 mol.%); however, the content of the copolymer did not exceed 40%. In this case, the indicator of bacterial growth was not the yield of cell biomass (X, g/L), but the optical density of the culture, which increased 10 times over 42 h of growth (from 0.2 to 27.0) at OD 600 nm).

In order to increase the content of 3MP monomers in P(3HB-*co*-3MP), the fractional feeding of 3-mercaptopropionic acid into a bacterial culture was studied. This original approach proposed by the authors of the paper earlier and implemented in a series of biotechnological processes, provided the synthesis of various types of PHA copolymers including those with high content of monomers other than 3-hydroxybutyrate. Modes of fractional and controlled supply of substrates-precursors of target monomers to a bacterial culture make it possible to avoid the effect of inhibition of bacteria. Moreover, regulated sampling at a certain time after the supply of precursors to a culture that is in a phase with a maximum rate of polymer synthesis and a minimum rate of endogenous biodegradation of PHA, as a rule, makes it possible to achieve a higher content of various monomers in the composition of the polymer chain [13,42,46,49,50,51].

**Figure 3:**
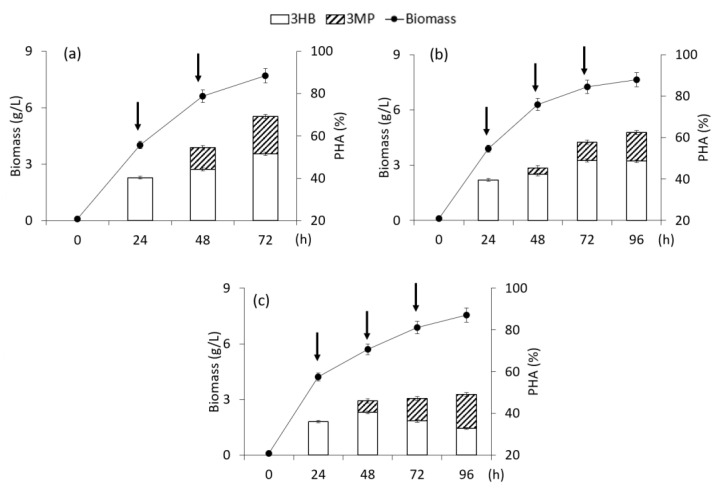
Biomass concentration of bacteria *Cupriavidus necator* B-10646, content and composition of PHA in experiments with fructose as the main C-substrate and addition of 3-mercaptopropionic acid at different concentrations: (**a**)—1.0 + 1.0; (**b**)—0.5 + 1.0 + 2.0; (**c**)—0.5 + 2.0 + 2.0 g/L; (the time of supplementation is shown by arrows).

The results of the influence of fractional dosing of 3-mercaptopropionic acid are shown in Figure 3.

A series of experiments included the supplementation of two and three additions of 3-mercaptopropionic acid into the culture at concentrations from 0.5 to 2.0 g/L. In the first experiment, the precursor substrate was added into the culture in the PHA synthesis mode at a concentration of 1.0 g/L for 24 h and 48 h of cultivation (total 2.0 g/L). In the second experiment, three additives of the precursor were added: 0.5 g/L for 24 h; 1.0 g/L—for 48 h, and 2.0 g/L—for 72 h (total 3.5 g/L); the cultivation time was increased to 96 h. In the third experiment, the first addition was 0.5 g/L, the second and third, 2.0 g/L each (4.5 g/L in total); the intervals between additions were 24 h (the cultivation time was increased to 96 h). The biomass concentration of *C. necator* B-10646 with fractional supply of the precursor substrate was practically the same as the values of 7.6 ± 0.1 g/L. However, the total content of the copolymer with an increase in the total amount of the precursor in the medium (from 2.0 to 4.5 g/L) showed a tendency to decrease from 69.2% to 49.0%. A similar effect of bacterial inhibition with an increase in the precursor concentration was described by other authors [22,23,31,33,36].

The fractional addition of 3-mercaptopropionic acid made it possible to obtain rather high inclusions of 3MP monomers in the copolymer composition, which was detected in the isolated polymer, from 7.6 mol.% to 16.0 mol.% and exceeded the values obtained with a single addition of 3-mercaptopropionic acid into the bacterial culture.

### 3.2. Study of 3,3′-Dithiodipropionic Acid as a Precursor for the Synthesis of Sulfur-Containing Copolymers in Cupriavidus necator B-10646 Culture

**Figure 4:**
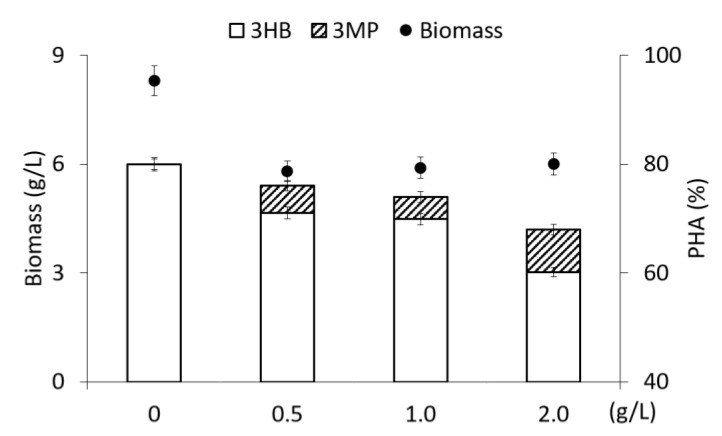
Biomass concentration of *Cupriavidus necator* B-10646, content and composition of PHA in experiments with fructose as the main C-substrate and addition of 3,3′-dithiodipropionic acid at various concentrations supplemented into medium on 24 h of cultivation.

3,3′-dithiodipropionic acid was studied as the second precursor. The biomass concentration of *C. necator* B-10646, the content and composition of PHA with a single addition of 3,3′-dithiodipropionic acid at a concentration from 0.5 to 2.0 g/L supplemented into the medium for 24 h of cultivation are given in Figure 4. The addition of this precursor to the minimum concentration (0.5 g/L) inhibited the growth of bacteria; the bacterial biomass yield decreased to 5.8 ± 0.2 g/L, significantly inferior to the values of the control experiment without the addition of the precursor (8.3 ± 0.2 g/L). With an increase in the concentration of 3,3′-dithiodipropionic acid in the medium, the inhibitory effect remained at the same level: when 2.0 g/L of the precursor was added, the concentration of bacterial biomass was 6.0 ± 0.2 g/L. This precursor did not inhibit polymer synthesis as significantly as bacterial growth. When the precursor was added at a concentration from 0.5 g/L to 1.0 g/L, the PHA yield remained at the level of 74.0 ± 1.0%, but when the concentration increased to 2.0 g/L, it slightly decreased to 68.0%. In these experiments, the detection of samples of the polymer isolated from bacterial cells showed an increase in the content of 3MP monomers from 6.59 mol.% to 11.53 mol.% with an increase in the concentration of the added precursor from 0.5 g/L to 2.0 g/L. This exceeds the performance achieved when using 3-mecaptopropionic acid as a precursor at similar concentrations by almost 2.5 times.

A similar inhibitory effect of 3,3′-dithiodipropionic acid as a precursor, but at a higher concentration (10.0 g/L), was observed by the authors [36,38]. They showed a decrease in the total intracellular content of the polymer (up to 19.0–35.0%) in bacterial cells of the wild-type strain *R. eutropha* H16. Unfortunately, the authors did not provide data on the yield of bacterial biomass in the paper. In the paper [37], the culture of *W. eutropha* H16 grown on sodium gluconate yields, in terms of bacterial biomass, were shown comparable to those obtained in this paper (about 6.0–7.0 g/L). In the papers [33,38], the content of 3MP monomers in the composition of poly(3HB-*co*-3MP) synthesized by the same *R. eutropha* H16 strain was higher (54.0–55.0 mol.%). However, significantly higher concentrations of the precursor (10.0 g/L) were used; it is not indicated what the yield of bacterial biomass was and which samples were analyzed—bacterial biomass or samples of the isolated and purified polymer. In another work [36], at a similar concentration of the precursor (10.0 g/L), the content of 3MP monomers was significantly lower (24.5 mol.%). It should be noted that in these works, despite the high content of 3MP monomers in copolymer, the total polymer content in bacterial cells was rather low (19.0–35.0%).

The effect of fractional feeding of 3,3′-dithiodipropionic acid into a bacterial culture on the biomass yield, total content and composition was studied. The precursor substrate was supplemented into the bacterial culture twice: at 1.0 g/L at 24 and 48 h of the bacterial cultivation process, this provided a cell biomass yield of 6.0 ± 0.1 g/L with a P(3HB-*co*-3MP) copolymer yield of 68.9% and the content of monomers 3MP, equal to 5.91 mol.%. Thus, it was not possible to achieve an increase in the content of 3MP monomers in the copolymer with fractional dosing of this precursor into the bacterial culture *C. necator* B-10646.

### 3.3. Study of 3,3′-Thiodipropionic Acid as a Precursor for the Synthesis of Sulfur-Containing Copolymers in the Culture of the Wild Strain C. necator B-10646

**Figure 5:**
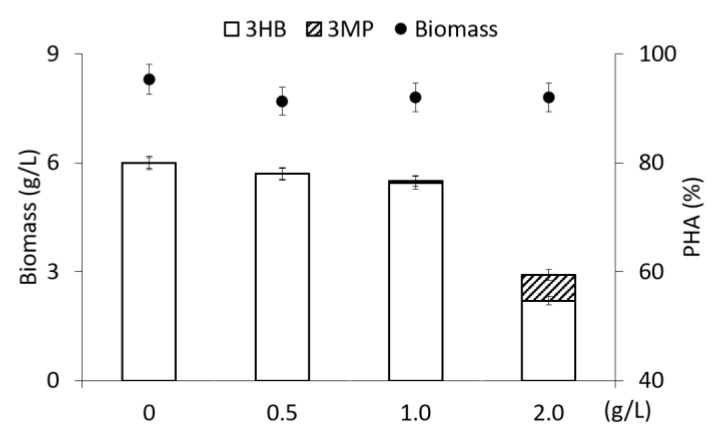
Biomass concentration of *Cupriavidus necator* B-10646, content and composition of PHA in experiments with fructose as the main C-substrate and addition of 3,3′-thiodipropionic acid at various concentrations supplemented into medium on 24 h of cultivation.

The third studied substrate-precursor for the synthesis of sulfur-containing PHA was 3,3′-thiodipropionic acid, at the first stage supplemented into the bacterial culture, as in previous experiments, once for 24 h of bacterial growth at a concentration of 0.5 to 2.0 g/L (Figure 5).

This precursor at concentrations of 0.5–1.0 g/L did not have a significant inhibitory effect on the yield of bacterial biomass and the overall yield of the polymer, which were close to those in the control. However, at these doses of the precursor, the inclusion of 3MP monomers in the copolymer composition was not fixed. An increase in the concentration of 3,3′-thiodipropionic acid to 2.0 g/L led to a halving of the biomass yield and the polymer content in the cells; in this case, the inclusion of 3MP monomers was very high (7.98 mol.%).

In the available literature, data on the content of sulfur-containing PHA when using this precursor are rather contradictory, while the production indicators of the culture in terms of the total yield of cell biomass are not reported. Thus, in the work [22], it was shown that when 3,3′-thiodipropionic acid was added to the culture of the wild-type strain *R. eutropha* H16 at a concentration of 2.0 g/L, the polymer content in the cells was low (19.2%). In the works of other authors [33,36], when using the same strain of bacteria at higher concentrations of 3,3′-thiodipropionic acid (9.0–10.0 g/L), the intracellular content of copolymer P(3HB-*co*-3MP) was higher (25.0–38.0%). Higher content of copolymer (up to 63.0%) at a similar concentration of 3,3′-thiodipropionic acid in culture were obtained by [38]. In all these works, fructose or gluconate was used as the main carbon source. In [39], sugars (fructose and glucose) and vegetable oils were used as the main carbon source for *C. necator* H16. The authors obtained higher concentrations of bacterial biomass (up to 6.3–7.3 g/L) and polymer content (29.0–46.0%) when 3,3′-thiodipropionic acid was added to the main carbon source (vegetable oils) at a concentration of 10.0 g/L. When sugars were used, biomass concentration and polymer content was lower and amounted to 4.1–4.2 g/L and 17.0–21.0%, respectively. The content of 3MP monomers in the copolymers varied from 4.0 mol.% to 27.0 mol.% and was inversely related to the intracellular content of the polymer.

**Figure 6:**
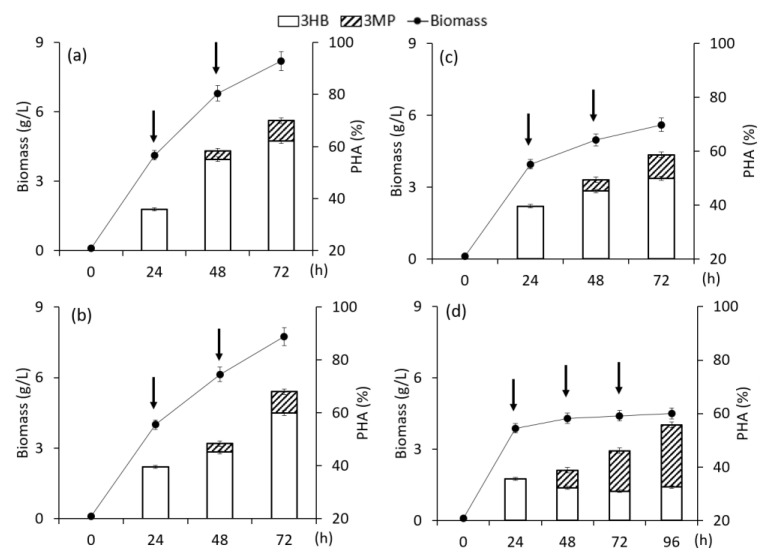
Biomass concentration of bacteria *Cupriavidus necator* B-10646, content and composition of PHA in experiments with fructose as the main C-substrate and addition of 3,3′-thiodipropionic acid acid at different concentrations: (**a**)—1.0 + 1.0; (**b**)—1.5 + 1.5; (**c**)—2.0 + 2.0; (**d**)—2.0 + 2.0 + 2.0 g/L; (the time of supplementation is shown by arrows).

Furthermore, the fractional introduction of 3,3′-thiodipropionic acid into the bacterial culture was studied (Figure 6).

A series of experiments included a fractional introduction of 3,3′-thiodipropionic acid in total concentrations from 2.0 to 6.0 g/L. In the first experiment, the precursor substrate was introduced into the culture in the PHA synthesis mode at a concentration of 1.0 g/L for 24 h and 48 h of bacterial cultivation (total 2.0 g/L). In the second experiment, the first addition (24 h of growth) was 1.5 g/L, the second addition (48 h of growth) was also 1.5 g/L (total 3.0 g/L). In the third experiment, the first and second additions were 2.0 g/L each (4.0 g/L in total) and in the fourth experiment three additions 2.0 g/L each (at 24 h, 48 h and 72 h, 6.0 g/L in total) were added; the cultivation time was increased to 96 h.

In experiments where a total amount of 2.0–3.0 g/L of 3,3′-thiodipropionic acid was introduced into the bacterial culture, the biomass yield was not inferior to the control (7.7–7.8 g/L). With an increase in the total concentration of the precursor to 4.0 and 6.0 g/L, a decrease in this indicator by one and a half times or more, to 5.6 g/L and 4.5 g/L, respectively. It is possible that it was caused by rather high doses of the precursor. With an increase in the total amount of 3,3′-thiodipropionic acid in the medium, a decrease in the total polymer content was noted, from 69.9% (with a total supply of a precursor of 2.0 g/L into the medium) to 52.4% (with a total supply of a precursor of 6.0 g/L). This mode of dosing of the precursor made it possible to achieve high inclusions of 3MP monomers in the copolymer P(3HB-*co*-3MP), up to 11.1 mol.%, 13.5 mol.% and 14.9 mol.% in experiments where the total supply of 3,3′-thiodipropionic acid was, respectively, 2.0 g/L, 3.0 g/L and 4.0 g/L. The maximum content of 3-mercaptopropionate monomers (up to 39.0 mol.%) was found at the highest concentration of the precursor (6.0 g/L). It is important to note that the obtained values reflect the actual content of 3MP in the composition of the copolymer, since samples of copolymers isolated from cell biomass and purified were analyzed. An analysis of publications showed (Table 1), that this precursor was studied for the synthesis of sulfur-containing PHA in a series of works [22,25,33,35,36,38,39]. So, in the culture of bacteria *R. eutropha* H16 and *C. necator* H16, depending on the type of carbon source (glucose, fructose, soya oil, safflower oil, castor oil or gluconate), the content of 3MP monomers varied very significantly and was minimally from 4.0 mol.% to 8.0 mol.%; maximally from 34.9 mol.% to 39.0 mol.%. This is comparable with the results presented in this work.

Thus, the use of 3,3-thiodipropionic acid as a precursor substrate made it possible to obtain a very high inclusion of 3MP monomers (up to 39.0 mol.%) in P(3HB-*co*-3MP) copolymers using the mode of fractional supply of the precursor to the culture.

### 3.4. Properties of Sulfur-Containing Copolymers P(3HB-co-3MP) Depending on the Ratio of Monomers

**Figure 7:**
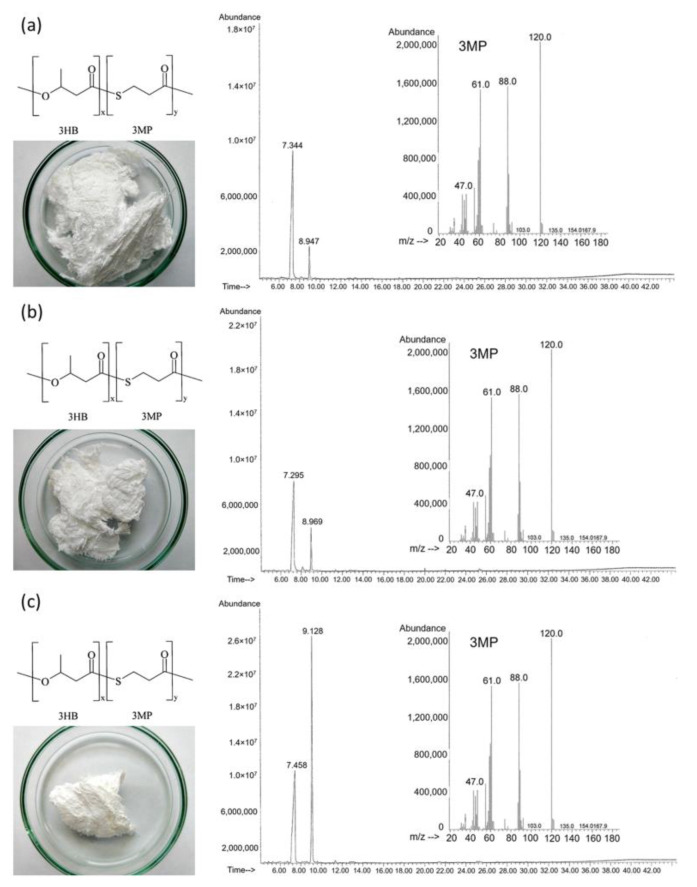
Photographs, structural formulas and chromatograms with mass spectra of the P(3HB-*co*-3MP) samples synthesized by *Cupriavidus necator* B-10646 using various 3MP monomer precursors: (**a**) 3-mercaptopropionic acid, the content of 3HB and 3MP monomers is 95.50 mol.% and 4.50 mol.%, respectively; (**b**) 3,3′-dithiodipropionic acid, the content of 3HB and 3MP monomers is 88.47 mol.% and 11.53 mol.%, respectively; (**c**) 3,3′-thiodipropionic acid, the content of 3HB and 3MP monomers is 61.0 mol.% and 39.0 mol.%, respectively. The retention time of the 3HB and 3MP monomers is 7.295–7458 and 8.947–9.128, respectively.

Regardless of the type of the studied substrate-precursor of 3MP monomers, all samples of PHA synthesized by *Cupriavidus necator* B-10646 were P(3HB-*co*-3MP) copolymers. Photographs of the synthesized copolymers P(3HB-*co*-3MP), structural formula and chromatograms are shown in Figure 7.

By varying the dosing regimen of precursors in the *C. necator* B-10646 culture, we made it possible to synthesize a series of P(3HB-*co*-3MP) copolymer samples with different ratios of 3-hydroxybutyrate (3HB) and 3-mercaptopropionate (3MP) monomers and to study the dependence of physicochemical properties of sulfur-containing copolymers (molecular weight and temperature characteristics and degree of crystallinity) on the ratio of monomers in P(3HB-*co*-3MP) (Table 2).

#### 3.4.1. Molecular Weight Characteristics of P(3HB-*co*-3MP) Copolymers

All studied P(3HB-*co*-3MP) samples, regardless of the type of precursor, had lower values of number average (M_n_) and weight average (M_w_) molecular weight and increased values of polydispersity (Ð) when compared to the P(3HB) homopolymer. In addition, it has been found that the molecular weight characteristics of P(3HB-*co*-3MP) copolymers are affected not only by the concentration of precursor substrates used, but also by the way they are dosed into the culture.

**Figure 8:**
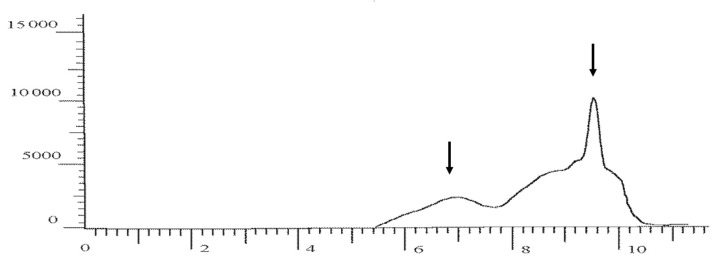
GPC chromatogram of a P(3HB-*co*-3MP) copolymer sample containing 10.8 mol.% of 3MP monomers and represented by two fractions with different molecular weights (marked with arrows).

Thus, with a single addition of 3-mercaptopropionic acid to a bacterial culture at a concentration of 0.7 g/L, the M_n_ and M_w_ values were 46 kDa and 202 kDa, respectively, and the polydispersity was 4.4. With an increase in the concentration of 3MP monomers to 2.0 g/L, the polydispersity of the polymer increased very significantly to 11.8, due to a decrease in M_n_ to 29 kDa. At a higher concentration of 3-mercaptopropionic acid (3.5 g/L) fed into the medium fractionally, the copolymer had higher M_n_ (73 kDa) and M_w_ (571 kDa) values, with the polydispersity value was still high (7.8). It was found that the sample No. 3 P(3HB-*co*-3MP) was synthesized by fractional addition of 3-mercaptopropionic acid (total concentration 4.5 g/L) to the culture is represented by two fractions with different values of molecular weights and polydispersity (Figure 8). The values of M_n_ for the low and high molecular weight polymer fractions were 35 kDa and 444 kDa, respectively. The ratio of these two fractions in the polymer was uneven; the low molecular weight polymer fraction dominated, amounting to 78%. Previously, we also showed that under certain conditions the synthesized polymer can be formed by two fractions with different molecular weight characteristics [52].

Similar reduced values of the molecular weight of the copolymer were found in the experiments with the other two precursors (3,3′-dithiodipropionic and 3,3′-thiodipropionic acids). With an increase in the concentration of 3,3′-dithiodipropionic acid from 0.5 g/L to 2.0 g/L, the M_n_ decreased from 67 kDa to 49 kDa and the polydispersity of the copolymer increased from 4.6 to 6.1 (samples No. 5–6 in Table 2). An increase in the concentration of 3,3′-thiodipropionic acid to 6.0 g/L was accompanied, as in the case of the other studied precursors, by an increase in polydispersity up to 10.7 (sample No. 10). It should be noted that a single addition of 3,3′-thiodipropionic acid at a concentration of 2.0 g/L had an effect on the molecular weight of the polymer to a lesser extent (M_n_ 157 kDa and M_w_ 471 kDa), in contrast to a similar concentration of 3-mercaptopropionic acid and 3,3′-dithiodipropionic acid.

The molecular weight and polydispersity of P(3HB-*co*-3MP) samples with different ratios of 3HB and 3MP monomers are the most studied aspect of the properties of these copolymers (Table 1). A comparison of the obtained results with the published ones showed that the literature data are not free from discrepancies and the molecular weight characteristics of the P(3HB-*co*-3MP) samples both coincide and differ with the data of this work. For example, in the works [22,31], the copolymer samples with close values to the content of 3MP monomers (40–42 mol.%) have very different values of M_w_ (190 and 790 kDa) and polydispersity (2.9 and 7.0), which are given (Table 1). In the work [33], at the same concentration of the 3,3′-dithiodipropionic acid and 3,3′-thiodipropionic acid, the M_w_ values for the copolymers differed and amounted to 150 kDa and 370 kDa, respectively. In the work [37], the M_w_ of a series of P(3HB-*co*-3MP) samples with different contents of 3MP monomers (from 16.0 mol.% to 38.0 mol.%) synthesized by *W. eutropha* H16 on sodium gluconate were also lower than that of P(3HB). However, at the same time, the M_w_ varied in a very wide range (150–606 kDa) at close values of polydispersity (3.1–4.4) without a direct relationship with the content of 3MP monomers. Similar to this work, the values of M_w_ (282–490 kDa) and polydispersity (2.8–4.2) for copolymer samples synthesized by *R. eutropha* H16 on fructose, on gluconate and on castor oil, respectively, are listed in [22,25,33,39]. In another paper [31], the lowest polydispersity value (1.1) was registered in the sample with the highest M_w_ value (1,120,000 kDa) and in samples with a reduced M_w_ value (about 600–700 kDa) the polydispersity was higher, at the level of 3–4. The M_w_ of P(3HB-*co*-3MP) samples synthesized in the culture of *R. eutropha* H16 on gluconate was very high, but different (minimum 760 kDa and maximum 1132 kDa). However, polydispersity of these copolymer samles was close (3.7–3.8) [35].

The results obtained in this work and comparisons with publications on the example of P(3HB-*co*-3MP) copolymers confirm the current ideas about the high variability of the molecular weight characteristics of PHA. The values of M_n_, M_w_ and polydispersity in the papers written by different authors have significant differences even when analyzing samples of similar chemical composition. This is likely due to the fact that the molecular weight is affected by many factors, including the type of microorganism-producer, cultivation conditions, type of carbon substrate, culture age and the method of extraction of the polymer from the cell biomass.

#### 3.4.2. Degree of Crystallinity of P(3HB-*co*-3MP) Copolymers

The most important characteristic of polymeric materials is the degree of crystallinity, which is the ratio of ordered (crystalline) and disordered (amorphous) phases. The ability of PHA of various chemical compositions to crystallize is determined by the internal properties of its chains and is characterized by the crystallization temperature (T_cryst_). In PHA, crystallization does not involve the entire volume of the material; therefore, these polymers are semi-crystalline objects. Studies of the dependence of the degree of crystallinity of copolymeric PHAs on the set and ratio of monomers in them is an insufficiently explored area. The study of the degree of crystallinity of sulfur-containing polymers is especially important in connection with the known data regarding the replacement of oxygen atoms in ether bonds by sulfur atoms in the polymer chain hinders the crystallization process [22].

**Figure 9:**
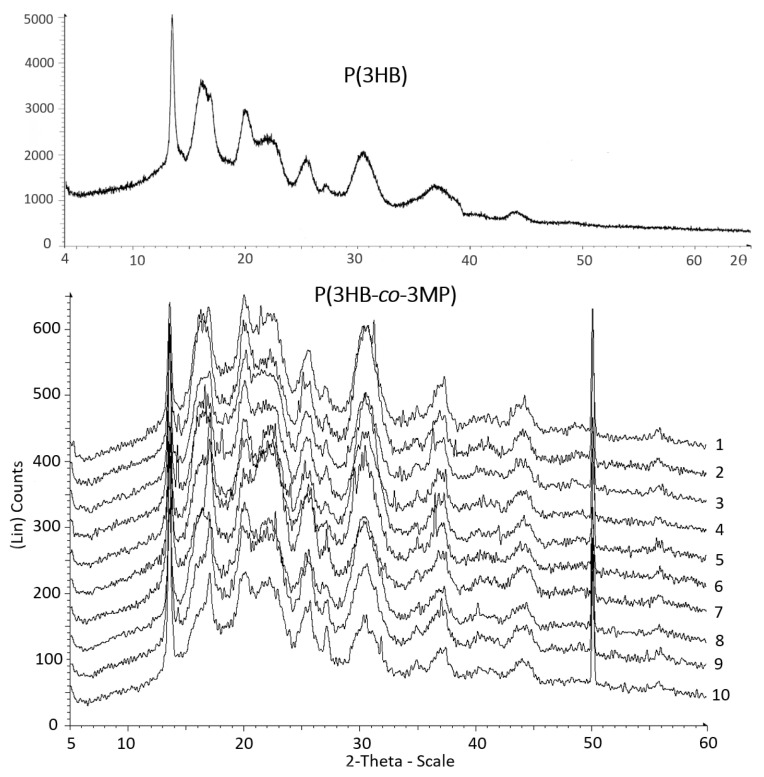
X-ray powder diffraction traces of P(3HB) and copolymers P(3HB-*co*-3MP) with different ratios of 3HB and 3MP monomers (sample numbering according to Table 2).

The results of X-ray diffraction analysis of a series of film samples of P(3HB-*co*-3MP) copolymers with different ratios of 3HB and 3MP monomers, in which the content of sulfur-containing 3MP monomers varied and was minimally 2.04 mol.% and maximally 39.0 mol.%, respectively (Table 2, Figure 9). Data on this indicator for P(3HB-*co*-3MP) copolymers are presented in this work for the first time since there are no quantitative indicators of the degree of crystallinity of sulfur-containing PHA in the available publications (Table 1).

Regardless of the ratio of monomers, all samples of copolymers had significantly reduced values of the degree of crystallinity (C_x_) compared with the homopolymer of conventional P(3HB) (74%). Despite the fact that the content of 3MP monomers varied by an order of magnitude in the P(3HB-*co*-3MP) samples, all of them had close ratios of the amorphous and crystalline phases. The lowest value of the degree of crystallinity (42%) was recorded for the sample with the highest content of 3MP monomers (39.0 mol.%). Higher C_x_ values (54.0; 51.0; and 50.0%) were recorded for samples with monomer content, respectively, 7.98 mol.%, 14.90 mol.% and 2.04 mol.%, i.e., without a clear relationship with the composition of monomers in P(3HB-*co*-3MP). Thus, it has been shown that insignificant inclusions of 3MP monomers in the composition of P(3HB-*co*-3MP) copolymers (even less than 10 mol.%) increases the proportion of the amorphous phase quite significantly.

All X-ray diffraction patterns of all samples of the P(3HB-*co*-3MP) copolymer showed the presence of a regular sharp diffraction peak at 2θ = 13.55°. This is similar to previously studied conventional PHAs that do not contain sulfur atoms in the polymer chain including P(3HB) homopolymer and copolymers containing, in addition to 3HB monomers, 3HB, 4HB and 3HHx monomers [13]. The second reflex in the form of a sharp diffraction peak fixed at 2θ = 50.12°. This value corresponds to the d-spacing equal to 1.82 Ǻ. Since this reflection is most likely of the second order, the value of the periodicity in the polymer is 3.64 Ǻ. This effect was discovered in this work for the first time in sulfur-containing P(3HB-*co*-3MP) copolymers and has not been described previously.

We did not find quantitative data on the degree of crystallinity of P(3HB-*co*-3HV) copolymers in the available literature. At the same time, in several works of colleagues from Germany, it was shown that the X-ray diffraction pattern of the poly(3HB-*co*-3MP) copolymer containing thioether bonds differs significantly from that of the P(3HB) homopolymer. X-ray diffraction patterns of samples with significantly different contents of 3MP monomers were taken and studied [31]. X-ray diffraction powder patterns of samples with the highest content of 3MP monomers (34.9 mol.%, 40.4 mol.% and 42.5 mol.%, respectively,) showed a sharp peak at 21.3°, 2θ which is not on X-ray diffraction patterns of P(3HB). However, it was absent in X-ray diffraction powder patterns of sample with the lowest content of monomers 3MP (4.6 mol.%) and the X-ray pattern completely coincided with that of P(3HB).

This shows that the crystallinity of P(3HB-*co*-3MP) can be affected by the content of 3MP monomers and this is the difference between the results of colleagues and those obtained in the present work, in which reflections were detected in other areas of the X-ray spectrum (at 2θ = 50.12°).

To reveal the regularities and mechanism of crystallization of sulfur-containing polymers, genetically engineered strains were obtained and studied, with the use of which it was possible to synthesize sulfur-containing homopolymers (PTEs): poly(3-mercaptopropionate) [(P(3MP)], poly(3-mercaptobutyrate) [P(3PMB)] and poly(3-mercaptovalerate) [P(3MV)] [32]. The X-ray powder diffraction traces of the sulfur-containing copolymers showed that the observed d-spacings for PTEs are different from those reported for equivalent oxygen analogues—“usual” PHAs (P(3HP), P(3HB) and P(3HV)) [24]. Moreover, the copolymer P(3HB-*co*-3MP) has a sharp diffraction peak at 21.3°, 2θ, which was absent in radiographs of samples for homooplymers of (P(3HB) and P(3HV) whose crystallinity is lower. The explanation of these differences is given in the works of colleagues from Germany. It was shown that the PHA family has ester dipole crystallization forces which are weaker in PTEs [32]. When a sulfur atom is inserted in the chain backbone, replacing an oxygen atom, crystallization may be hindered because the electronegativity of sulfur (2.58) is much lower than that of oxygen (3.44) [24]. Sulfur’s value is the same as that for carbon (2.55). As shown in work [53] in the case of P(3HB), ester dipole interactions stabilize its helical conformation and when sulfur atoms replace oxygen they may not have this favorable Coulombic influence on helix stability. This hinders crystallization and the polymer is amorphized.

#### 3.4.3. Temperature Characteristics of P(3HB-*co*-3MP) Copolymers

The temperature characteristics of PHA and the ability to crystallize in the native state are significant parameters, since they determine the thermomechanical properties and, consequently, the possibility of processing into products from melts. In PHA, similarly to many polymeric materials, the temperature at which deformation occurs is somewhat lower than the boiling point (thermal degradation temperature); therefore, the gas state in polymers is not realized and the main type of phase equilibrium in them is the condensed state—crystalline, glassy, viscous and liquid. The presence of a pronounced range between the temperature of the onset of melting and the temperature of the onset of thermal decomposition is an important technological property of the polymer, since it makes it possible to obtain products based on it by conventional methods of processing polymer melts (solution molding, extrusion and injection molding).

**Figure 10:**
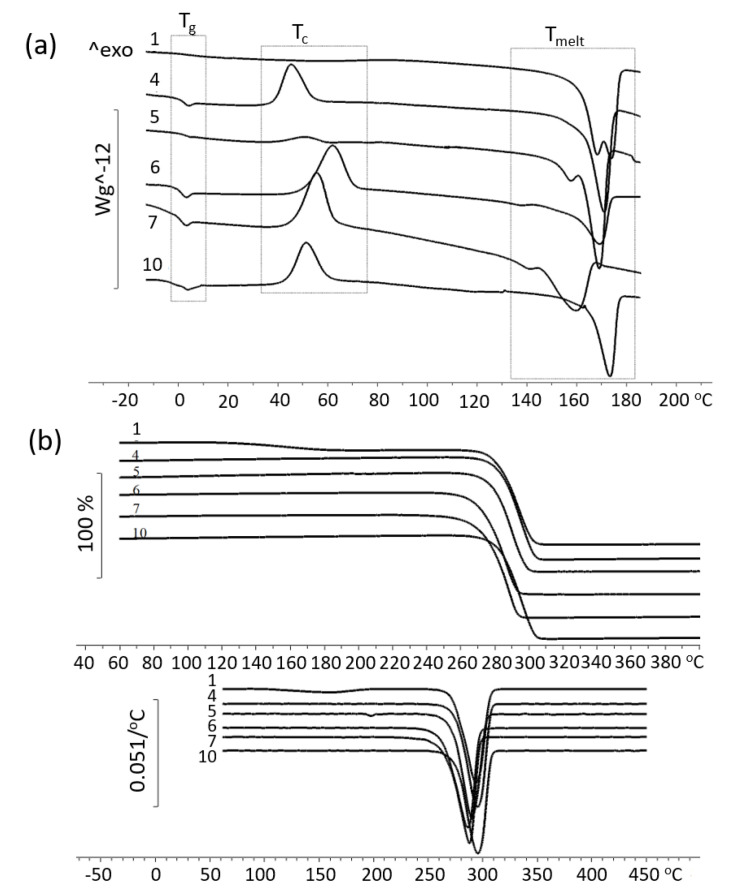
Temperature characteristics of P(3HB-*co*-3MP) samples with different sets of monomers: (**a**) DSC curves with glass transition temperature (T_g_), crystallization temperature (T_cryst_) and melting point (T_melt_) regions; (**b**) thermal stability (TGA) (sample numbering according to Table 2).

The results of studying the temperature characteristics of the synthesized P(3HB-*co*-3MP) samples are illustrated in Table 2 and Figure 10.

The recorded values of the melting point (T_melt_) and thermal degradation temperature (T_degr_) for most of the studied samples are comparable with the T_melt_ and T_degr_ for P(3HB) and only for some samples these values decreased (Figure 10a). Attention is drawn to the presence of two peaks in some of the samples, both in the region of melting and thermal degradation with different discontinuities in the temperature intervals between the peaks. At the same time, for a large part of samples with 3MP monomer content from 2.04 to 11.53 the gap between two peaks ranged from 5.2 °C to 32.4 °C without a direct relationship with the content of sulfur-containing monomers. A similar effect of the presence of two peaks in the melting region was also shown in [39] for P(3HB-*co*-3MP) samples with different contents of 3MP monomers (from 7.0 mol.% to 22.0 mol.%), while the values of the region melting points were somewhat lower than those of the samples in this work, and those of P(3HB). Similar results on the left-shift melting behavior of P(3HB-*co*-3MP) copolymers with different 3MP monomer contents are presented in [31,37]. Lütke-Eversloh showed that P(3HB-*co*-3MP) copolymers with a monomer content from 4.5 mol.% to 42.5 mol.%, in contrast to our data, generally have lower melting points (not higher than 111–123 °C) (Table 1); however, the glass transition and crystallization temperatures are close to the values obtained in our work [31].

All P(3HB-*co*-3MP) samples are characterized by a decrease in the glass transition temperature (T_g_) with an increase in the inclusion of 3MP monomers (Table 2). The published data on the temperature characteristics of P(3HB-*co*-3MP) copolymers are far from being an exhaustive picture. As shown in Table 1, the majority of publications have data on the melting point of these copolymers. At the same time, information on the temperature of thermal degradation is almost completely absent; data on crystallization and glass transition temperatures are given for a few samples. Comparison of the results of the dependence of the crystallization temperature on the content of 3MP monomers in the copolymer obtained in this work is consistent with the data of other authors [35,37]. The maximum and minimum T_g_ values were recorded for samples obtained using 3-mercaptopropionic acid as a precursor. The highest glass transition temperature was sample No. 2 with 3MP 2.04 mol.% (T_g_ 9.9 °C). The lowest T_g_ was recorded for sample No. 4 with 3MP content 10.8 mol.% (T_g_ −14.5 °C). For this sample, as in the case of the molecular weight distribution, two glass transition temperatures were recorded, which indicates the presence of two fractions in the sample. For all samples obtained using 3,3′-dithiodipropionic acid, 3,3′-thiodipropionic acid and sample No. 4 obtained using 3-mercaptopropionic acid as an increase in the glass transition step is characteristic and after that step, a small peak appears, indicating relaxation processes in molecules. The 3MP indicates an increase in the amorphous phase, which is confirmed by the data of X-ray diffraction analysis.

The melting point of all samples obtained using 3-mercaptopropionic acid as a precursor was in the range of 167.0–173.5 °C. Samples with the content of 3MP 2.04 mol.% and 10.8 mol.% have a bifurcation of the melting peaks. For a sample containing 4.50 mol.% of 3MP the second peak appears as a shoulder on the main peak. The effect of 3MP monomers on the melting point was not revealed. These data are consistent with those obtained in [31], when 3-mercaptopropionic acid was used as a precursor. The authors also found double and even triple melting peaks and no relationship between the 3MP incorporation and the melting point. It should be noted that the samples of sulfur-containing copolymers obtained in the present work had higher melting points than the samples synthesized by Lütke-Eversloh and collegues.

Samples prepared using 3,3′-dithiodipropionic acid as a precursor had lower melting points than the samples described above. Melting points varied in a wide range from 137 °C to 169 °C. Thermograms are also characterized by double melting peaks. In this case, an increase in the width of the melting peaks should be noted. It may indicate the formation of several phases in the polymer with different melting rates. It was noted that the width of the melting peak increased as the 3MP monomers in the copolymers increased. Thus, a sample with the 6.59 mol.% of 3MP melted in the range 150–172 °C and a sample with a content of 3MP 11.53 mol.% melted in the range 124–173 °C. In general, the melting point values obtained by us for these samples are consistent with the data given in [35], but are slightly higher than for the samples presented in [37].

For the melting points of P(3HB-*co*-3MP) samples synthesized using 3,3′-thiodipropionic acid, we found that an increase in 3MP monomers in copolymers leads not only to an increase in the melting point, but also to a decrease in the interval in which samples are melted. Thus, a sample containing 2.23 mol.% of 3MP monomers melted in the temperature range from 130 °C to 166 °C, with two peaks at 141 °C and 159 °C, respectively, and these are the minimum temperatures for the studied series of samples. Samples containing 14.9 mol.% and 39.0 mol.% of 3MP monomers melted in the temperature range from 161 °C to 176 °C and had single, narrow melting peaks at 171 °C and 173 °C, respectively. The presence of narrower and single melting peaks may indicate a greater homogeneity of the resulting crystals. The temperature indices obtained for these samples are somewhat inconsistent with those published. However, it should be noted that information on the temperature characteristics of sulfur-containing PHAs is limited.

Crystallization of samples obtained using 3-mercaptopropionic acid as a precursor, containing 2.0 mol.% and 4.5 mol.% of 3MP monomers in their composition, occurred upon cooling T_cryst_ to 69.5 °C and 88.5 °C, respectively. It is typical for the crystallization of P(3HB) homopolymer. A sample containing 10.8 mol.% of 3MP monomers is characterized by the presence of three crystallization peaks. This suggests that each fraction in the copolymer has its own crystallization temperature range. Primary crystallization of fractions occurs upon cooling T_cryst_ to 57.7 °C and 83.9 °C, respectively. With further heating, pre-crystallization or recrystallization occurs at a temperature of 37.6 °C, which may be due to an increase in the mobility of parts of the molecules included in the amorphous part of the sample. As in the case of the previous sample, the sample containing 10.8 mol.% of 3MP monomers has two crystallization peaks (at 69.8 °C during cooling and at 45.2 °C during subsequent heating). This behavior of the crystalline part of the samples may be due to an increase in the amorphous component in the polymers. There is also information in the literature about the lack of correlation between the crystallization temperature and the 3MP monomer content [31].

A sample containing 6.59 mol.% of 3MP monomers and prepared using 3,3′- dithiodipropionicacid as a precursor also had two crystallization peaks. One part of the crystal is formed during cooling at a temperature of 61 °C and further crystallization of the sample occurs during reheating at a temperature of 51 °C. All other samples obtained using both 3,3′-dithiodipropionic acid and 3,3′-thiodipropionic acid under thermal analysis conditions did not show crystallization peaks upon cooling. It may indicate difficult crystallization and requiring a long time for crystallization. Crystallization of the samples was recorded upon reheating. The minimum T_cryst_ of 49 °C was recorded for a sample with a 3MP monomer content of 7.98 mol.%. For the rest of the samples, T_cryst_ was in the range of 51–62 °C. When the samples are cooled, the mobility of the amorphous part of the molecules decreases, the so-called “freezing”, which in turn prevents the development of the crystalline phase due to spatial restrictions because one polymer molecule can participate both in the formation of a crystal and be in the amorphous part of the polymer. With further heating of the sample, the “frozen” part of the molecule devitrifies and the mobility of this part increases; it can take part in further completion of the already formed crystalline phase or the formation of a new one. It should be noted that there is insufficient information in the literature on the crystallization temperatures of sulfur-containing P(3HB-*co*-3MP) copolymers obtained using 3,3′-thiodipropionic acid as a precursor.

The thermal stability of the obtained samples was evaluated by thermogravimetry (Figure 10b). The lowest thermal stability was recorded for samples obtained using 3-mercaptopropionic acid with different content of 3MP monomers (2.04 mol.% and 10.8 mol.%). For a sample with a content of 2.04 mol.% of 3MP monomers, degradation of a part of the sample (by approximately 7.7%) was recorded at a temperature of 132.9 °C. For a sample with a 3MP content of 10.8 mol.%, 2.8% of the mass degrades at 138.7 °C. The degradation of the rest of the molecules in these samples was recorded at 279 °C and 281 °C, respectively. The reduced thermal stability of the samples, apparently, can be explained by the low molecular weight and complex fractional composition with different molecular weight distributions which negatively affected the thermal stability. The rest of the P(3HB-*co*-3MP) samples studied showed high thermal stability. T_degr_ was in the range from 257 °C to 282 °C. The gap between the melting and degradation temperatures ranged from 88 °C 115 °C.

The performed experiments were the first to study in detail the patterns of synthesis of sulfur-containing P(3HB-*co*-3MP) copolymers with various content of 3MP monomers; a family of copolymers with different ratios of 3MP monomers was synthesized and new results on the effect of the composition of sulfur-containing P(3HB-*co*-3MP) copolymers on the physicochemical properties were obtained.

## 4. Conclusions

Modes of cultivation of wild-type strain *C. necator* B-10646 with fractional addition of various precursor substrates: 3-mercaptopropionic acid, 3,3′-dithiodipropionic and 3,3′-thiodipropionic acids were implemented. By varying the concentration and number of doses of the precursor supplemented into the bacterial culture, it was possible to find conditions that ensure the formation of 3MP monomers from the precursor and their incorporation into the C-chain of poly(3-hydroxybutyrate). A set of copolymers with different contents of 3-hydroxybutyrate (3HB) monomers (from 60.10 mol.% to 97.96 mol.%) and inclusions of 3-mercaptopropionate (3MP) monomers (from 2.04 mol.% to 39.0 mol.%) were synthesized. The polymer was purified to a homogeneous state suitable for determining the true chemical composition. Using high-performance liquid chromatography, differential thermal analysis, differential scanning calorimetry and X-ray diffraction analysis, studies have been carried out for the first time that have revealed a relationship between the composition of P(3HB-*co*-3MP) copolymers and the physicochemical properties of them. It was shown that new types of monomers (3MP) into PHA affect the molecular weight, temperature characteristics and the degree of crystallinity of the synthesized copolymers. An important result of the influence of new monomers on the properties of PHA was obtained, which consists of the alignment of the amorphous and ordered phases and a significant decrease in the degree of crystallinity (below 50%) of the synthesized copolymer samples. A decrease in the degree of crystallinity of PHA samples has a positive effect on their properties and crystallization kinetics, which facilitates processing into specialized products, improving technological properties.

## Figures and Tables

**Table 1:** Literature data on synthesis and properties of sulfur-containing copolymers when using various precursors for the synthesis of 3MP monomers.

Strain, Substrate	X, g/L	PHA, %	3HB, mol.%	3MP, mol.%	M_w_, kDa	Đ	T_melt_, °C	T_degr_, °C	T_g_, °C	T_cryst_, °C	C_x_, %	Reference
Precursor of 3-mercaptopropionic acid												
*R. eutropha* H16, gluconic acid	-	11.4	57.5	42.5	790	7.0	-	-	-	-	-	[22]
*R. eutropha* H16, sodium gluconate	-	-	59.6	40.4	190	2.9	123.4/ 164.9	-	−1.7	71.0	-	[31]
	-	-	57.5	42.5	790	7.0	111.1/ 159.2	-	-	64.4	-	
	-	-	73.1	26.9	490	4.2	102.2/ 137.5/ 153.9	-	−3.3	62.6	-	
	-	-	65.1	34.9	1120	1.1	111.6/ 132.6/ 168.6	-	-	50.3	-	
	-	-	89.7	10.3	886	1.2	173.2	-	-	-	-	
	-	-	74.9	25.1	132	2.1	150.2/ 163.9	-	-	-	-	
	-	-	84.2	15.8	470	3.5	155.0/ 163.2	-	-	56.9	-	
	-	-	92.8	7.2	715	1.8	151.3/ 163.2	-	-	59.1	-	
	-	-	95.4	4.6	633	2.7	164.2	-	-	52.2	-	
	-	-	91.3	8.7	749	3.6	162.9	-	-	52.3	-	
Precursor of 3,3′-dithiodipropionic acid												
*R. eutropha* H16, gluconate	-	18.6	46.0	54.0	150	3.1	-	-	-	-	-	[33]
*R. eutropha* H16, gluconate	-	33.1	75.5	24.5	-	-	-	-	-	-	-	[36]
*W. eutropha* H16, sodium gluconate	7.45	8.1	71.9	28.1	314	4.2	111.9/ 129.1	-	−3.0	-	-	[37]
	6.71	2.9	61.4	38.6	498	3.69	56.3/ 153.0	-	−3.2	-	-	
	6.73	9.7	52.0	48.0	251	3.3	55.1/ 149.1	-	−10.7 /0.5	-	-	
	7.16	17.3	80.6	19.4	443	4.43	144.6	-	2.0	-	-	
	5.09	20.2	83.3	16.7	606	2.1	144.2	-	1.5	-	-	
*R. eutropha* H16, sodium gluconate	-	34.0	51.0	49.0	-	-	-	-	-	-	-	[38]
Precursor of 3,3′-thiodipropionic acid												
*R. eutropha* H16, fructose	-	19.2	73.1	26.9	490	4.2	-	-	-	-	-	[22]
*R. eutropha* H16, gluconate	-	9.2	65.1	34.9	1120	1.1	-	-	-	-	-	
*R. eutropha* H16, gluconate	-	-	82.0	18.0	792	2.4	-	-	-	-	-	[25]
*R. eutropha* H16, gluconate	-	24.8	77.0	23.0	370	2.8	-	-	-	-	-	[33]
*R. eutropha* H16, gluconate	-	-	94.0	6.0	1132	3.7	170.0	288	3.0	58.0	-	[35]
	-	-	86.0	14.0	999	3.7	168.0	-	−16.3	52.0	-	
	-	-	78.0	22.0	760	3.8	161.0	286	−17.2	64.0	-	
*R. eutropha* H16, gluconate	-	38.4	84.9	15.1	-	-	-	-	-	-	-	[36]
*R. eutropha* H16, sodium gluconate	-	57.0	89.4	10.6	-	-	-	-	-	-	-	[38]
*C. necator* H16, castor oil	6.33	28.7	83.0	17.0	282	4.1	-	-	-	-	-	[39]
	-	-	93.0	7.0	-	-	137.7/ 154.4	-	1.7	-	-	
	-	-	61.0	39.0	-	-	143.3/ 165.4	-	−4.7	-	-	
	-	-	78.0	22.0	-	-	132.3/ 151.7	-	−0.5	-	-	
	-	-	88.0	12.0	-	-	136.4/ 156.3	-	1.6	-	-	
	-	-	68.0	32.0	-	-	137.2/ 152.6	-	−3.1	-	-	
*C. necator* H16, safflower oil	6.89	35.0	88.0	12.0	-	-	-	-	-	-	-	
*C. necator* H16, rice oil	7.30	42.5	92.0	8.0	-	-	-	-	-	-	-	
*C. necator* H16, corn oil	6.27	41.4	87.0	13.0	-	-	-	-	-	-	-	
*C. necator* H16, soya oil	6.82	46.1	96.0	4.0	-	-	-	-	-	-	-	
*C. necator* H16, peanut oil	7.06	33.7	90.0	10.0	-	-	-	-	-	-	-	
*C. necator* H16, olive oil	6.87	46.4	92.0	8.0	-	-	-	-	-	-	-	
*C. necator* H16, fructose	4.19	20.9	84.0	16.0	-	-	-	-	-	-	-	
*C. necator* H16, glucose	4.06	17.3	75.0	25.0	-	-	-	-	-	-	-	

- information is absent.

**Table 2:** Physicochemical properties of P(3HB-*co*-3MP) with different ratio of monomers.

N	Precursor Concentration, g/L	Composition of Monomers, mol.%		M_n_, kDa	M_w_, kDa	Ð	C_x_, %	T_melt_ (°C)	T_degr_ (°C)	T_g_ (°C)	T_c_ (°C)
		3HB	3MP								
3-mercaptopropionic acid precursor											
1	0.7	97.96	2.04	46	202	4.4	50	167.9 173.5	132.9 279.3	9.9	69.5
2	2.0	95.50	4.50	29	342	11.8	48	169.2	257.1	5.3	88.5
3	4.5 (fractionally)	89.20	10.80	120 (22%) 12 (78%)	444 (22%) 35 (78%)	3.7 (22%) 2.9 (78%)	45	168.0 173.2	138.7 281.1	−14.5 1.6	57.7 83.9 37.6
4	3.5 (fractionally)	83.96	16.04	73	569	7.8	47	170.7	280.9	1.4	69.8 45.2
3,3′-dithiodipropionic acid precursor											
5	0.5	93.41	6.59	67	308	4.6	48	157.4 168.7	277.5	0.8	60.9 51.0
6	2.0	88.47	11.53	49	299	6.1	47	136.6 169.0	270.1	−0.56	61.7
3,3′-thiodipropionic acid precursor											
7	2.0	97.77	2.23	157	471	3.0	50	141.0 159.3	271.2	−0.8	55.5
8	2.0 (fractionally)	92.02	7.98	80	336	4.2	54	143.3 168.7	282.5	−0.1	48.7
9	4.0 (fractionally)	85.10	14.90	37	337	9.1	51	171.4	280.1	−0.9	61.3
10	6.0 (fractionally)	61.00	39.00	32	342	10.7	42	173.1	280.6	−3.3	51.2
P(3HB)											
11	0	100	0	368	920	2.5	74	179.3	279.4	5.3	81.6

## Data Availability

All data is available in the paper.
